# The influence of axial myopia on optic disc characteristics of glaucoma eyes

**DOI:** 10.1038/s41598-021-88406-1

**Published:** 2021-04-23

**Authors:** Jasmin Rezapour, Christopher Bowd, Jade Dohleman, Akram Belghith, James A. Proudfoot, Mark Christopher, Leslie Hyman, Jost B. Jonas, Massimo A. Fazio, Robert N. Weinreb, Linda M. Zangwill

**Affiliations:** 1 Hamilton Glaucoma Center, Shiley Eye Institute, Viterbi Family Department of Ophthalmology, University of California, San Diego, 9500 Gilman Drive, La Jolla, CA 92093-0946 USA; 2 Department of Ophthalmology, University Medical Center of the Johannes Gutenberg University, Mainz, Germany; 3 Wills Eye Hospital, Thomas Jefferson University, Philadelphia, PA USA; 4 Department of Ophthalmology, Medical Faculty Mannheim, Heidelberg University, Mannheim, Germany; 5 Department of Ophthalmology and Vision Science, School of Medicine, The University of Alabama At Birmingham, Birmingham, AL USA; 6 Department of Biomedical Engineering, School of Engineering, The University of Alabama At Birmingham, Birmingham, AL USA

**Keywords:** Optic nerve diseases, Refractive errors

## Abstract

This study characterizes differences in glaucomatous eyes with and without high axial myopia using custom automated analysis of OCT images. 452 eyes of 277 glaucoma patients were stratified into non (n = 145 eyes), mild (n = 214 eyes), and high axial myopia (axial length (AL) > 26 mm, n = 93 eyes). Optic disc ovality index, tilt and rotation angle of Bruch´s membrane opening (BMO) and peripapillary choroidal thickness (PCT) were calculated using automated and deep learning strategies. High myopic optic discs were more oval and had larger BMO tilt than mild and non-myopic discs (both *p* < 0.001). Mean PCT was thinnest in high myopic eyes followed by mild and non-myopic eyes (*p* < 0.001). BMO rotation angle, global retinal nerve fiber layer (RNFL) thickness and BMO-minimum rim width (MRW) were similar among groups. Temporal RNFL was thicker and supranasal BMO-MRW was thinner in high myopic eyes. BMO tilt and PCT showed moderate and temporal RNFL and nasal BMO-MRW showed weak but significant associations with AL in multivariable analyses (all *p* < 0.05). Large BMO tilt angle and thin PCT are characteristics of highly myopic discs and were not associated with severity of glaucoma. Caution should be exercised when using sectoral BMO-MRW and RNFL thickness for glaucoma management decisions in myopic eyes.

## Introduction

There is consistent evidence that the prevalence of myopia has markedly increased. Persons with high myopia are also more likely to have primary open angle glaucoma than non-highly myopic individuals. Specifically, the prevalence of myopia is increasing from approximately 1.4 billion worldwide in 2010, to an estimated 5 billion by 2050^1^. In addition, a meta-analysis of 7 studies suggests individuals with myopia are 2.5 times more likely to have glaucoma than those without myopia^2^. It is unclear why myopia increases the risk of glaucoma, but it is likely related to a greater susceptibility of the optic nerve head to glaucoma damage through a variety of mechanisms. For example, it has been discussed that a thinner lamina cribrosa in myopic eyes makes it more susceptible due to a steepening of the pressure gradient^3^, and combined with secondary enlargement of the optic nerve head from myopia and elongation and thinning of the peripapillary scleral flange, it may increase an individual’s risk for developing glaucoma^3–6^. Moreover, the diagnosis of glaucoma often is challenging in high myopes as the appearance of the myopic optic nerve head, with its higher degree of tilt and possibly oval shape and torsion mimics glaucomatous optic disc damage making the diagnosis of glaucoma difficult in high myopes^7–10^. It follows that the myopic optic disc may mistakenly be interpreted as glaucoma resulting in possible overdiagnosis and overtreatment of glaucoma in non-glaucomatous myopes^11^. Hence, the study of morphological features of the myopic optic disc can improve our understanding of the relationship between myopia and glaucoma.

In the majority of studies, ovality index, disc tilt and torsion measurements have been evaluated based on qualitative review of fundus photographs relative to the clinical disc margin. These studies report inconsistent results with respect to the association of these parameters with axial length^7–9,12–18^. The problem with a photograph-based approach to measure the ovality index, disc tilt or torsion is that landmarks such as the clinical disc margin are subjective and not based on the actual three-dimensional anatomy of the optic disc^19–21^. Furthermore, there is evidence that with increasing axial length the Bruch’s membrane opening (BMO) anatomy is less likely to be represented by the clinical disc margin so that a two-dimensional photography-based assessment does not reflect the true disc configuration in all aspects^22^. An advantage of using optical coherence tomography (OCT) imaging to measure ovality index, tilt and torsion is that an objective identification and measurement of these features relative to the BMO, is possible^23^.

The objective of this study is to improve our understanding of the morphological characteristics of the glaucomatous optic disc in axial highly myopic and non-myopic eyes. We measured standard optic disc parameters including retinal nerve fiber layer (RNFL) thickness and BMO-MRW (minimum rim width) as well as partially novel features, BMO tilt, BMO torsion, BMO ovality and peripapillary choroidal thickness (PCT) based on custom image processing and deep learning analysis of OCT scans relative to the BMO.

## Methods

### Participants

This cross-sectional study included all glaucoma patients enrolled in the Diagnostic Innovations in Glaucoma Study (DIGS; clinicaltrials.gov identifier NCT00221897) with axial length, visual field measurements and good quality spectral domain (SD)-OCT of the optic nerve head (ONH) using radial circle (RC) scans. The institutional review board of the University of California San Diego approved the study methods, which are in accordance with HIPAA regulations and according to the Declaration of Helsinki, written informed consent was obtained from all participants. A detailed description of the study design has been published previously^24^. Subjects were ≥ 18 years old and had open anterior chamber angles. All participants received a complete ophthalmologic examination including refractometry and assessment of best-corrected visual acuity, standard automated perimetry (Humphrey Field Analyzer; 24–2 Swedish interactive thresholding algorithm [SITA] standard; Carl-Zeiss Meditec), Goldmann applanation tonometry, gonioscopy, dilated fundus examination, central corneal thickness (CCT) measurement by ultrasound pachymetry (DGH Technology, Inc., Exton, PA), coherence interferometry measurement of the axial length (IOLMaster, Carl Zeiss Meditec, Dublin, CA), and simultaneous stereophotography and SD-OCT of the optic disc and macula.

The definition of primary open angle glaucoma was based on the DIGS conventional “gold standard” of visual field loss and photograph-based optic disc damage^24^. Stereo photograph-based optic disc damage was defined as focal or diffuse narrowing of the neuroretinal rim, and/or detection of retinal nerve fiber layer (RNFL) defects characteristic of glaucoma as based on a masked assessment by two trained observers. Two experts (JR and CB) graded photographs after a high myopia optic disc grading training with a senior consultant (JBJ) with expertise in myopia and glaucoma. Stereo photograph-based optic disc damage was defined by consensus between both graders. In case of disagreement, diagnosis was defined by adjudication by the senior consultant.

The Visual Field Assessment Center (VisFACT) Reading Center completed the quality control of all visual fields according to standard protocols and unreliable visual fields were excluded^24^. Standard automated perimetry glaucomatous visual field damage was defined as two repeatable and reliable visual field tests (rate of fixation losses and false negatives and false positives responses of < 33%) with a glaucoma hemifield test (GHT) outside normal limits and/ or a pattern standard deviation (PSD) with a *p* value of < 0.05 with a similar defect on consecutive abnormal tests^24^.

### Axial myopia categories

Axial elongation can lead to morphological changes of the optic disc and the fundus^25,26^. Myopia defined by the refractive error does not always imply axial elongation that can lead to the morphological changes mentioned above. At the same time cataract surgery or refractive procedures might lead to a refractive change in eyes that are axially elongated but do no longer appear to be (highly) myopic as defined by refractive error. This is also confirmed by the distribution of the spherical equivalent in our study population (Table 1). This study assessed the morphological changes of the optic disc in association with axial length and for these reasons myopia was defined by axial length and not by refractive error. Based on population-based studies^2,27^, axial myopia groups were defined as follows:

- No axial myopia: axial length ≤ 24.0 mm
- Mild axial myopia: 24.0 mm < axial length ≤ 26.0 mm
- High axial myopia: axial length > 26.0 mm

**Table 1:** Glaucoma patient and eye characteristics by myopia group.

	No myopia 81 (145 eyes)	Mild myopia 128 (214 eyes)	High myopia 68 (93 eyes)	*p* value (age-adjusted)
Age	77.2 (74.7, 79.6)	73.0 (71.1, 74.9)	65.6 (62.6, 68.7)	< 0.001^1, 2, 3^
Sex (% Female)	65.4%	43.0%	47.1%	0.005^1, 2^
**Race**				
European descent	67.9%	68.8%	66.2%	0.410
African descent	23.5%	17.2%	13.2%	
Asian descent	7.4%	12.5%	17.6%	
Other/unknown	1.2%	1.6%	2.9%	
AL (mm)	23.4 (23.3, 23.5)	24.8 (24.7, 24.9)	26.7 (26.5, 26.8)	< 0.001^1, 2, 3^ (< 0.001^1, 2, 3^)
^a^SE (D)	0.21 (− 0.24, 0.66)	− 1.36 (− 1.74, − 0.98)	− 3.49 (− 4.06, − 2.92)	< 0.001^1, 2, 3^ (< 0.001^1, 2, 3^)
^b^CCT (µm)	533.6 (525.4, 541.8)	534.5 (527.7, 541.4)	529.6 (519.4, 539.9)	0.788 (0.774)
VF MD (dB)	− 6.09 (− 7.41, − 4.77)	− 6.90 (− 7.99, − 5.81)	− 7.34 (− 8.91, − 5.76)	0.450 (0.083^2^)
^c^IOP (mmHg)	14.1 (13.2, 15.1)	13.9 (13.1, 14.7)	13.3 (12.1, 14.4)	0.514 (0.046^2^)
^d^BMO area (mm^2^)	2.11 (2.01, 2.20)	2.12 (2.04, 2.20)	2.18 (2.06, 2.30)	0.608 (0.954)

Results are presented as mean (95% confidence interval) or percentage. Race was compared using a chi-squared test. Continuous variables were compared using ANOVA (for age) or linear mixed models (for eye level data). No myopia: AL ≤ 24.0 mm; Mild myopia: AL: > 24 mm and ≤ 26.0 mm; High myopia: AL > 26.0 mm.

Missing 66^a^, 14^b^, 2^c^, and 28^d^ values.

^1^No versus Mild Myopia *p* < 0.05; ^2^No versus High Myopia *p* < 0.05; ^3^Mild versus High Myopia *p* < 0.05.

AL, axial length; BMO, Bruch’s membrane opening; CCT, central corneal thickness; IOP (intraocular pressure); SE, spherical equivalent; VF MD, visual field mean deviation.

### Image acquisition

The Spectralis OCT was used to obtain 24 high-resolution optic nerve head (ONH) radial scans and 3 retinal nerve fiber layer (RNFL) circle scans centred on the optic nerve head using the ONH radical circle (ONHRC) scan from the Glaucoma Module Premier Edition software (version 6.10; Heidelberg Engineering Inc, Heidelberg, Germany). Heidelberg Engineering Spectralis standard software was used to calculate BMO area, BMO-MRW and RNFL thickness. Images were electronically transferred for quality assessment to the Imaging Data Evaluation and Analysis (IDEA) Center. OCT images were reviewed for accurate BMO segmentation in the radial B-scans and accurate internal limiting membrane (ILM), RNFL and Bruch’s membrane (BM) segmentation in the circle scans. If needed, these automated segmentations were manually corrected according to standard IDEA Reading Center protocols. Poor quality images (quality score < 15) or those with BMO segmentation failure that could not be manually corrected were excluded.

The BMO-MRW, defined as the shortest distance from the BMO point to the ILM^28^, was automatically computed by instrument software for each radial scan and averaged for all 24 scans. Circumpapillary RNFL thickness was measured from the 3 circle scans obtained at approximately 3.5 mm, 4.1 mm and 4.7 mm diameter using instrument software. For this study RNFL thickness measurements were based on the 3.5 mm diameter circle scan.

### Measurement of optic disc BMO ovality, tilt and rotation

**Figure 1:**
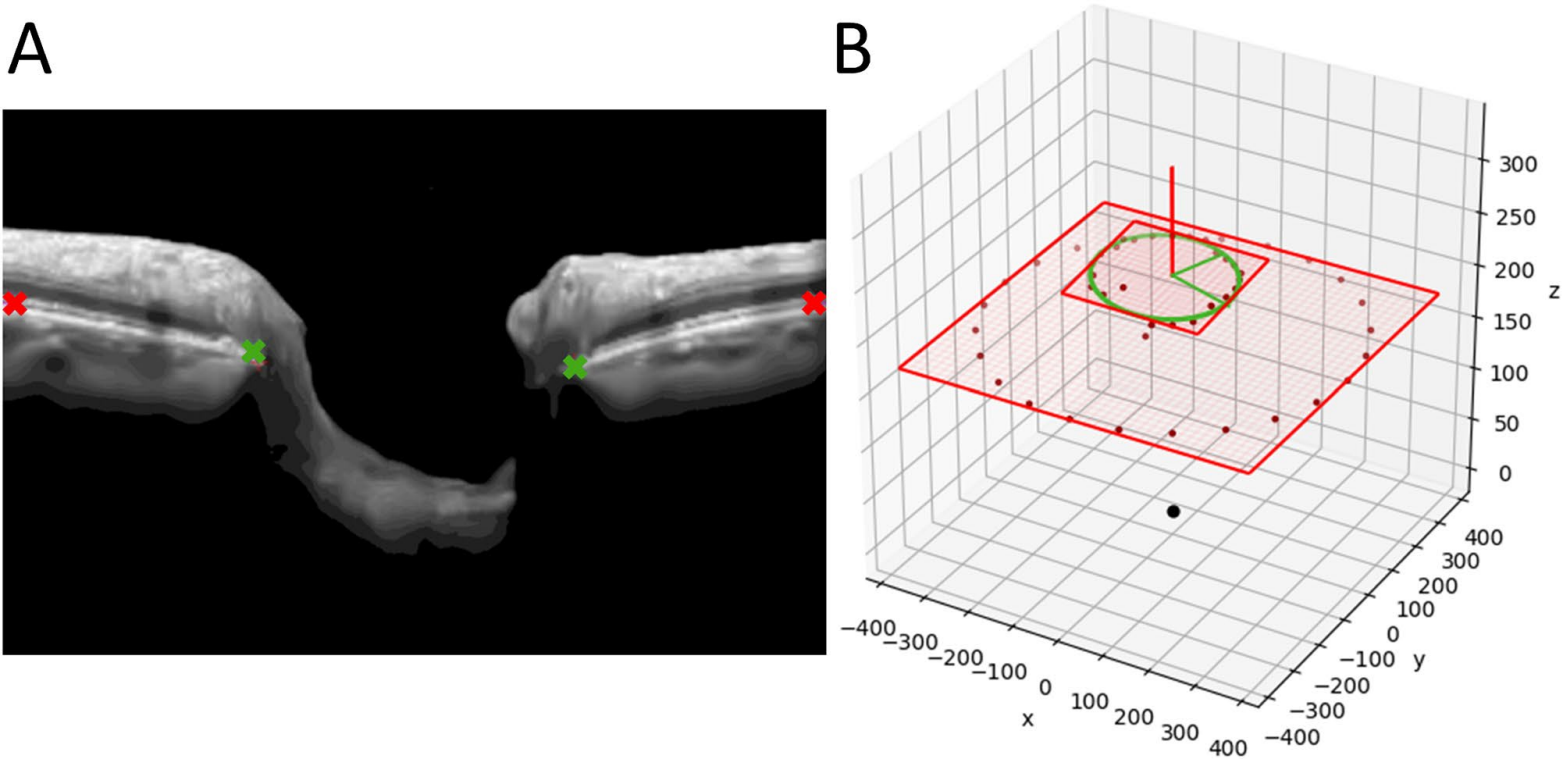
3D rectangular coordinate space illustrating the BMO fit plane, the BMB fit plane and the corresponding BMB- and BMO points derived from OCT B-scans to calculate tilt and ovality index. (**A**) SALSA-segmented optic disc B-scan demonstrating 2 BMO points (green) and 2 BMB points (red) used to project on the rectangular coordinate space to calculate BMO ovality index, tilt and rotation angle. (**B**) The green ellipse represents the fit BMO ellipse and the smaller red rectangle represents the fit BMO plane. The larger red rectangle represents the fit BMB plane, calculated from the BMB ellipse (red dots inside the larger red rectangle). The red vector from the center of the fit ellipse represents the normal vector to the fit ellipse and helps to illustrate the tilt. The black point represents the origin of the coordinate system. The two green vectors from the center of the fit ellipse are the semi-major and semi-minor axes used to calculate the BMO ovality index calculated as the minor BMO axis divided by the major BMO axis. Abbreviations: BMB, Bruch’s membrane boundary; BMO, Bruch’s membrane opening; SALSA, San Diego Automated Layer Segmentation Algorithm.

Optic disc ovality, tilt and rotation were automatically calculated on the BMO points using custom San Diego Automated Layer Segmentation Algorithm (SALSA) software^29^, as Spectralis software does not provide these measurements. It should be noted that the custom calculations were not based on the clinical disc margin but on the BMO. With this novel method we utilize the term BMO rotation^19^ instead of using the more commonly used term disc “torsion”. By using “rotation” we aim to characterize the purely anatomical characteristics of the BMO-based disc margin, contrary to “torsion”, which implies shearing deformations of the peripapillary and ONH tissue, which can only be proven by histological examinations. To calculate these three parameters, two BM boundary (BMB) points of the BM at the edges of each of the 24 radial B-scan (furthest peripheral pixels on each BM edge of a B-scan in total two BMB points per B-scan and two BMO points per B-scan were calculated (Fig. 1A). Details of the calculations are provided in the Supplemental Material.

The ovality index was calculated by dividing the shortest diameter of the BMO by the longest diameter. The major and minor axes were defined as the longest and shortest diameter of the fitted BMO ellipse.

BMO tilt angle (measured in degrees) was defined as the angle between BMO plane and BMB plane normal vectors. After transforming the coordinates in the 3D rectangular space, one 2D plane was fit to the BMB points and one to the BMO points using linear least squares method (Fig. 1B). BMO tilt angle was calculated by assessing the angle between their normal vectors.

**Figure 2:**
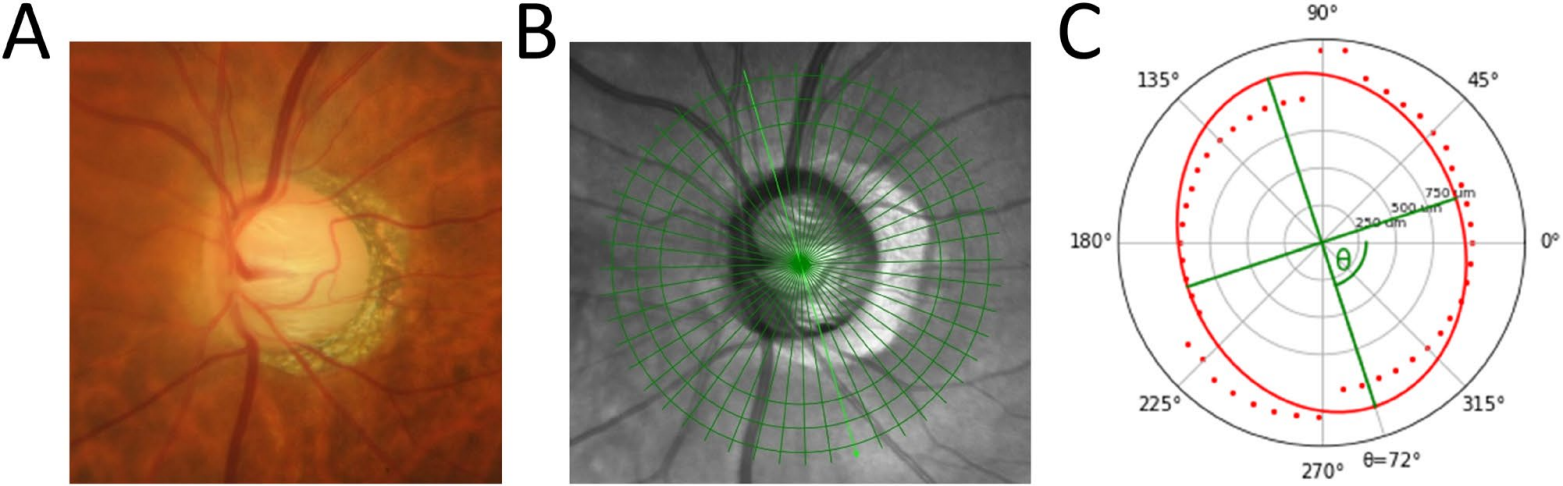
Case illustration of BMO ovality index (0.82) and BMO rotation angle (72.4°) measurement in a highly myopic left eye (AL = 26.1 mm). The photograph (**A**) and OCT infrared image (**B**) of the optic disc are shown on the left and the corresponding BMO ovality and rotation measurement (**C**) is illustrated on the right image. The red dots indicate the BMO points. The red ellipse represents the fit ellipse based on the BMO points. The green vectors indicate the shortest and longest diameter of the BMO ellipse and are defined as major and minor axis to calculate the BMO ovality index. The BMO rotation angle is defined as the angle between the semimajor axis and the temporal axis (0°, horizontal axes of the en face OCT image), adjusted for variation in individual anatomies based on the FoBMOc angle. Abbreviations: BMO, Bruch’s membrane opening; FoBMOc, Fovea BMO center; OCT, optical coherence tomography.

To calculate the BMO rotation angle (measured in degrees), each BMO point was projected onto the BMO reference plane and used to fit a two-dimensional ellipse to the points in the reference plane. The orientation and ovality of the fit ellipse was used to compute the BMO rotation angle and ovality index, respectively (Fig. 2).

### BMO ovality, tilt and rotation validation

Automated BMO ovality index measurement was validated manually (JR) for reliability in a randomly chosen subset of 50 non-, mild- and high axial myopic eyes and showed good performance. To validate the BMO tilt and rotation measurements described above we independently assessed both the segmentation and quantification algorithms by reprocessing the ONHRC scans with a commercially available auto-segmentation software (ReflectivityTM; AbyssProcessing, Singapore)^30^ and then calculating the same 3D morphological parameters described in the methods with a custom algorithm based developed by MAF in Mathematica (Wolfram Research, Urbana, Illinois). It should be noted that the cross-validation method used a fixed distance of 1700 µm from the center of the BMO to determine the reference points for the tilt calculation.

### Measurement of peripapillary choroidal thickness using SALSA-deep

As Spectralis software does not provide choroidal thickness measurements, custom software (SALSA-deep) was developed using a deep learning strategy, BCDU-Net^29^ to segment the RNFL circle scan (3.5 mm diameter) of the ONHRC (Supplemental Figure 2) to identify the BM and the posterior boundary of the choroid to calculate choroidal thickness. Details of how BCDU-Net an extension of U-Net, bi-directional ConvLSTM was applied is available in the Supplemental Material. Peripapillary choroidal thickness (PCT) was defined as the perpendicular distance between the posterior border of the BM and the posterior boundary of the choroid. Global and sectoral PCT was calculated for temporal, superotemporal, inferotemporal, nasal, superonasal, and inferonasal sectors. The automated deep learning strategy was articulated as follow: First, a trained grader (JR) manually segmented the BM and the posterior boundary of the choroid in circle scans of the ONHRC scan in the Spectralis software (version 6.10; Heidelberg Engineering Inc, Heidelberg, Germany). This subset of eyes was used as the ground truth to train a BCDU-Net deep convolutional neural network model. In total, 385 eyes of 190 non axial myopic, mild axial myopic subjects, and 15 eyes of 10 high axial myopic subjects were used to train the model. Scans with poor quality (quality score < 15 dB) were excluded from the segmentation process.

The automated deep learning BCDU-Net based choroidal segmentation of each RNFL circle scan was reviewed for accuracy (JR). The RNFL scans of 21 eyes were not available for segmentation due to low image quality. The overall performance of the deep learning segmentation algorithm was very good with 379/384 (98.7%) scans considered accurately segmented and included in the analysis. In addition, the proportion of eyes with good quality choroidal segmentation was over 95% in each of the myopia groups (no myopia: 135/139 [97.1%], mild myopia: 203/207 [98.1%] and high myopia: 41/43, [95.4%]).

### Statistical analyses

Statistical analysis was conducted using categorical data to compare between axial myopia groups and continuous data to analyze an association between different ocular parameters and axial length. Data is presented as mean (95% confidence interval) and count (percentage) for continuous and categorical variables, respectively. The statistical significance of comparisons between patient-level characteristics across myopia groups was determined by analysis of variance (ANOVA) for continuous variables and chi-squared tests for categorical variables. For eye-level characteristics, mean and confidence interval estimates were derived from linear mixed effects models, with a random intercept to account for within-subject correlation. Overall significance across myopia category was determined from these models using F-tests, with denominator degrees of freedom computed via Satterthwaite’s method. Significance of pairwise comparisons of myopia groups was determined similarly by t-tests of regression parameters with degrees of freedom calculated via Satterthwaite’s method. Univariate and age and VF MD adjusted models were fit to evaluate the association between axial length and ocular parameters of interest. Similar univariate and multivariate (adjusted for age and axial length) regression models were fit to evaluate the association between VF MD and ocular parameters. R-squared values were computed via the methods of Edwards el. al, with partial R-squared computed for the fixed effect of interest in age and VF MD multivariate models^31^. Pearson correlation coefficient was applied to assess correlation between BMO ovality index, BMO tilt and BMO rotation. All statistical analyses were performed using the R (version 3.5.2)^32^. We consider *p* values less than 0.05 to indicate statistical significance throughout.

## Results

Four hundred fifty-two glaucoma eyes of 277 patients were included with 145 eyes (81 patients) in the no axial myopia group, 214 eyes (128 patients) in the mild axial myopia group and 93 eyes (68 patients) in the high axial myopia group (Table 1). Mean (95% [CI]) age (years) in the high axial myopia group was significantly younger (65.6 [62.6, 68.7]) compared to the mild (73.0 [71.1, 74.9]) and no axial myopia (77.2 [74.7, 79.6]) group (*p* < 0.001). The proportion of individuals of Asian descent was higher (17.6%) and of African descent lower (13.2%) in the high axial myopia group, compared to the mild and no axial myopia group (*p* = 0.410). Mean (95% [CI]) spherical equivalent was significantly lower in high axial myopes (− 3.49 [− 4.06, − 2.92]) compared to mild (− 1.36 [− 1.74, − 0.98]) and non-myopes (0.21 [− 0.24, 0.66]) (age adjusted *p* < 0.001). There was no significant difference in mean visual field mean deviation (MD) (*p* = 0.450), IOP (*p* = 0.514), central corneal thickness (CCT) (*p* = 0.788) and BMO area (age adjusted *p* = 0.608) between the three groups.

### OCT optic disc parameters

A total of 21 eyes were excluded from the analysis for not meeting image quality criteria of ONHRC scans (quality score > 15 dB or ONHRC segmentation failure) with 9/145 (6.2%), 7/214 (3.3%) and 5/46 (10.9%) eyes excluded from the no-, mild- and high axial myopia group, respectively. Excluded eyes were significantly younger (mean age = 62.7 years, *p* = 0.009), had a higher axial length (mean = 25.1 mm, *p* = 0.032) and a worse VF MD (mean = −14.3 dB, *p* = 0.001) than included eyes.

BMO morphometrics (ovality index and tilt angle) and choroidal thickness differed by axial myopia group. BMO rotation angle, global and sectoral RNFL thickness values and global and sectoral BMO-MRW values except in the temporal sector for the RNFL and the superonasal sector for the BMO-MRW did not differ significantly between the axial myopia groups. These results along with age- and VF MD adjusted *p* values are presented in Table 2. Specifically, the BMO of high axial myopes was significantly more oval (0.85 [0.84, 0.87]) compared to no axial myopes (0.88 [0.86, 0.89]) and mild axial myopes (0.89 [0.88, 0.90]; *p* < 0.005), but this difference is small and may not be clinically important. The BMO also was significantly more titled in high myopes (3.4° [3.1, 3.8]) compared to mild myopes (2.0° [1.8, 2.2]) and no myopes (1.7° [1.5, 2.0]; *p* < 0.001). In contrast to global and other sectoral RNFL and BMO-MRW thickness values, in multivariable analysis in the high myopia group temporal RNFL thickness was significantly thicker and the superonasal BMO-MRW was significantly thinner compared to the no myopia group.

**Table 2:** Optic disc parameters by myopia group.

	No myopia	Mild myopia	High myopia	*p* value (Age and VF MD adjusted *p* value)
**BMO morphometrics**	77 (128 eyes)	118 (185 eyes)	51 (61 eyes)	
Ovality index	0.88 (0.86, 0.89)	0.89 (0.88, 0.90)	0.85 (0.84, 0.87)	0.003^2, 3^ (0.007^3^)
Tilt angle (°)	1.7 (1.4, 2.0)	2.0 (1.8, 2.2)	3.4 (3.1, 3.8)	< 0.001^2, 3^ (< 0.001^2, 3^)
Rotation angle (°)	36.1 (32.6, 39.7)	35.0 (32.1, 37.9)	36.7 (31.4, 42.0)	0.818 (0.803)
**RNFL thickness (µm)**	81 (145 eyes)	128 (214 eyes)	68 (93 eyes)	
Global	67.1 (64.2, 70.0)	65.2 (62.8, 67.6)	67.5 (64.0, 70.9)	0.427 (0.419)
Temporal	52.6 (49.7, 55.5)	54.6 (52.2, 56.9)	58.8 (55.4, 62.3)	0.024^2, 3^ (0.029^2, 3^)
Superotemporal	85.9 (80.9, 90.9)	81.5 (77.4, 85.7)	85.2 (79.2, 91.3)	0.332 (0.284)
Inferotemporal	89.9 (83.6, 96.1)	82.8 (77.6, 87.9)	89.2 (81.7, 96.7)	0.135 (0.139)
Nasal	57.6 (54.8, 60.4)	58.0 (55.7, 60.3)	57.7 (54.3, 61.1)	0.972 (0.934)
Superonasal	77.4 (73.0, 81.7)	72.2 (68.6, 75.8)	71.4 (66.1, 76.7)	0.122 (0.120)
Inferonasal	73.4 (69.0, 77.9)	68.1 (64.5, 71.7)	70.3 (65.1, 75.6)	0.162 (0.227)
**BMO-MRW (µm)**	80 (142 eyes)	124 (204 eyes)	59 (78 eyes)	
Global	199.1 (188.8, 209.4)	192.4 (183.8, 201.0)	188.0 (174.8, 201.2)	0.387 (0.392)
Temporal	148.9 (140.4, 157.5)	148.3 (141.1, 155.4)	155.6 (144.6, 166.6)	0.513 (0.784)
Superotemporal	179.4 (166.4, 192.3)	162.6 (151.8, 173.4)	170.9 (154.2, 187.5)	0.128^1^ (0.102^1^)
Inferotemporal	182.4 (167.6, 197.3)	177.2 (164.7, 189.6)	178.5 (159.4, 197.7)	0.856 (0.945)
Nasal	226.2 (212.7, 239.7)	228.8 (217.5, 240.0)	211.1 (193.7, 228.5)	0.226 (0.262)
Superonasal	226.9 (213.9, 239.9)	206.6 (195.7, 217.5)	192.0 (175.3, 208.8)	0.004^1, 2^ (0.003^1, 2^)
Inferonasal	243.9 (228.9, 258.9)	224.2 (211.7, 236.7)	217.0 (197.6, 236.4)	0.052^1, 2^ (0.121)
**Choroidal thickness (µm)**	76 (135 eyes)	126 (203 eyes)	47 (61 eyes)	
Global	135.8 (125.4, 146.2)	122.3 (113.9, 130.8)	109.8 (96.4, 123.1)	0.006^1, 2^ (< 0.001^1, 2, 3^)
Temporal	140.8 (129.0, 152.6)	121.0 (111.5, 130.6)	95.0 (79.6, 110.5)	< 0.001^1, 2, 3^ (< 0.001^1, 2, 3^)
Superotemporal	152.5 (140.3, 164.6)	136.6 (126.8, 146.5)	114.1 (98.2, 130.0)	0.001^1, 2, 3^ (< 0.001^1, 2, 3^)
Inferotemporal	106.5 (97.2, 115.8)	95.1 (87.5, 102.6)	82.7 (70.6, 94.8)	0.007^1, 2^ (< 0.001^1, 2, 3^)
Nasal	142.7 (130.9, 154.4)	131.4 (121.8, 140.9)	128.6 (113.3, 144.0)	0.207 (0.029^1, 2^)
Superonasal	152.8 (140.2, 165.4)	140.9 (130.7, 151.1)	128.7 (112.1, 145.2)	0.062^2^ (0.003^1, 2^)
Inferonasal	111.2 (101.3, 121.1)	100.7 (92.7, 108.7)	100.0 (87.0, 112.9)	0.181 (0.020^1, 2^)

Results are presented as mean (95% confidence interval). Significance is determined by linear mixed models. No myopia: AL ≤ 24.0 mm; Mild myopia: AL: > 24 mm and ≤ 26.0 mm; High myopia: AL > 26.0 mm.

BMO-MRW, Bruch’s membrane opening minimum rim width; ONH, optic nerve head; RNFL, retinal nerve fiber layer; VF MD, visual field mean deviation.

^1^No versus Mild Myopia *p* < 0.05; ^2^No versus High Myopia *p* < 0.05; ^3^Mild versus High Myopia *p* < 0.05.

Global and sectoral PCT values were also significantly thinner in highly axial myopic eyes compared to mild axial myopic eyes and non-axial myopic eyes. The sectoral thickness pattern of the PCT was similar across no-, mild- and high myopic groups with the PCT thinnest in the inferotemporal sector (106.5 µm vs. 95.1 µm vs. 82.7 µm, respectively) and thickest in the superonasal sector (152.8 µm vs. 140.9 µm vs. 128.7, respectively) (Table 2).

**Figure 3:**
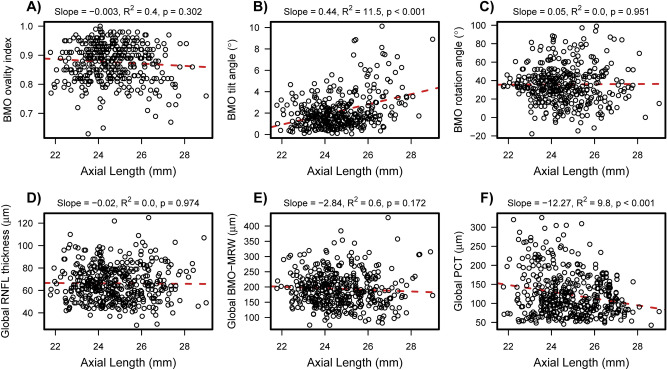
Scatter plots demonstrating association between axial length and ocular characteristics. Significant albeit relatively weak associations were found between axial length and BMO tilt angle (**B**) and PCT (**F**) in multivariate (adjusted for age and VF MD) regression analysis. No significant associations were found between axial length and BMO ovality index (**A**), BMO rotation angle (**C**), global RNFL thickness (**D**) and global BMO-MRW (**E**), respectively. Abbreviations: BMO, Bruch’s membrane opening; MRW, minimum rim width; PCT, peripapillary choroidal thickness; RNFL, retinal nerve fiber layer.

Not all optic nerve head parameters that differed across myopic groups were significantly associated with axial length (Fig. 3). Specifically, in multivariable models adjusted for age and VF MD (Table 3), there was a significant (all *p* < 0.001) but weak to moderate linear association (semi-partial R^2^) between axial length and the BMO tilt angle (R^2^ = 10.4%) and global (R^2^ = 8.5%) and sectoral PCT with R^2^ values ranging from 4.0% in the nasal sector to 15.8% in the temporal sector. BMO ovality index (R^2 =^0.4%, *p* = 0.289), BMO rotation angle (R^2^ = 0.0%, *p* = 0.947), global RNFL thickness (0.0%, *p* = 0.966) and BMO-MRW (0.3%, *p* = 0.294) were not significantly associated with axial length in linear models. However, a weak, significant association between axial length and RNFL thickness in the temporal sector (R^2^ = 2.1%, *p* = 0.006, respectively) and BMO-MRW in the superonasal and inferonasal sectors (R^2^ = 1.9%, *p* = 0.012 and R^2^ = 1.2%, *p* = 0.046, respectively) was found.

**Table 3:** The association of ocular characteristics with axial length.

	N	Univariate regression^a^		Multivariable regression Age and VF MD adjusted^a^	
	Patients (Eyes)	Estimate (95% CI)	R^2^ (*p* value)	Estimate (95% CI)	Semi-partial R^2^ (*p* value )
**Age**	277 (452)	− 0.37 (− 0.63, − 0.11)	0.3 (0.006)	− 0.37 (− 0.63, − 0.11)	0.3 (0.005)
**VF MD**	277 (452)	− 0.30 (− 0.84, 0.23)	0.4 (0.262)	− 0.64 (− 1.20, − 0.08)	1.4 (0.026)
**BMO morphometrics**	246 (374)				
Ovality index		− 0.004 (− 0.009, 0.001)	0.7 (0.135)	− 0.003 (− 0.009, 0.003)	0.4 (0.289)
Tilt angle (°)		0.45 (0.33, 0.57)	13.4 (< 0.001)	0.42 (0.29, 0.55)	10.4 (< 0.001)
Rotation angle (°)		− 0.18 (− 1.78, 1.42)	0.0 (0.829)	− 0.06 (− 1.76, 1.65)	0.0 (0.947)
**RNFL thickness (µm)**	277 (452)				
Global		− 0.04 (− 1.21, 1.13)	0.0 (0.946)	0.02 (− 1.05, 1.10)	0.0 (0.966)
Temporal		1.70 (0.53, 2.87)	2.3 (0.005)	1.71 (0.49, 2.93)	2.1 (0.006)
Superotemporal		− 0.32 (− 2.37, 1.72)	0.0 (0.756)	− 0.36 (− 2.22, 1.50)	0.0 (0.707)
Inferotemporal		− 0.98 (− 3.51, 1.55)	0.2 (0.448)	− 0.50 (− 2.80, 1.80)	0.1 (0.670)
Nasal		0.01 (− 1.12, 1.15)	0.0 (0.981)	0.02 (− 1.15, 1.19)	0.0 (0.968)
Superonasal		− 1.29 (− 3.05, 0.47)	0.6 (0.151)	− 1.40 (− 3.20, 0.39)	0.6 (0.127)
Inferonasal		− 1.54 (− 3.32, 0.24)	0.8 (0.092)	− 1.34 (− 3.09, 0.41)	0.6 (0.135)
**BMO-MRW (µm)**	263 (424)				
Global		− 1.95 (− 6.31, 2.41)	0.2 (0.382)	− 2.22 (− 6.37, 1.92)	0.3 (0.294)
Temporal		2.69 (− 0.92, 6.30)	0.7 (0.146)	1.73 (− 1.92, 5.39)	0.3 (0.353)
Superotemporal		− 1.79 (− 7.28, 3.70)	0.1 (0.524)	− 3.31 (− 8.44, 1.81)	0.5 (0.206)
Inferotemporal		− 0.21 (− 6.50, 6.07)	0.0 (0.948)	− 0.59 (− 6.51, 5.33)	0.0 (0.846)
Nasal		− 2.78 (− 8.52, 2.96)	0.3 (0.344)	− 2.16 (− 8.05, 3.72)	0.2 (0.472)
Superonasal		− 6.78 (− 12.29, − 1.27)	1.7 (0.017)	− 6.97 (− 12.38, − 1.56)	1.9 (0.012)
Inferonasal		− 7.09 (− 13.40, − 0.77)	1.4 (0.029)	− 6.26 (− 12.39, − 0.13)	1.2 (0.046)
**Choroid thickness (µm)**	249 (399)				
Global		− 9.29 (− 14.00, − 4.58)	5.0 (< 0.001)	− 12.93 (− 17.61, − 8.25)	9.1 (< 0.001)
Temporal		− 15.31 (− 20.58, − 10.04)	10.3 (< 0.001)	− 19.67 (− 24.89, − 14.46)	15.8 (< 0.001)
Superotemporal		− 11.91 (− 17.38, − 6.44)	6.1 (< 0.001)	− 15.91 (− 21.37, − 10.44)	10.0 (< 0.001)
Inferotemporal		− 8.99 (− 13.18, − 4.81)	5.9 (< 0.001)	− 12.57 (− 16.72, − 8.43)	10.8 (< 0.001)
Nasal		− 5.99 (− 11.29, − 0.68)	1.7 (0.028)	− 9.46 (− 14.78, − 4.13)	4.0 (< 0.001)
Superonasal		− 7.67 (− 13.36, − 1.99)	2.4 (0.009)	− 11.53 (− 17.24, − 5.82)	5.1 (< 0.001)
Inferonasal		− 4.81 (− 9.26, − 0.36)	1.6 (0.035)	− 8.09 (− 12.57, − 3.62)	4.1 (< 0.001)

BMO-MRW, Bruch’s membrane opening minimum rim width; CI, confidence interval; ONH, optic nerve head; RNFL, retinal nerve fiber layer; VF MD, visual field mean deviation.

^a^Linear mixed models slope estimates (with 95% confidence intervals) from univariate and multivariable models adjusted for age and VF MD.

**Figure 4:**
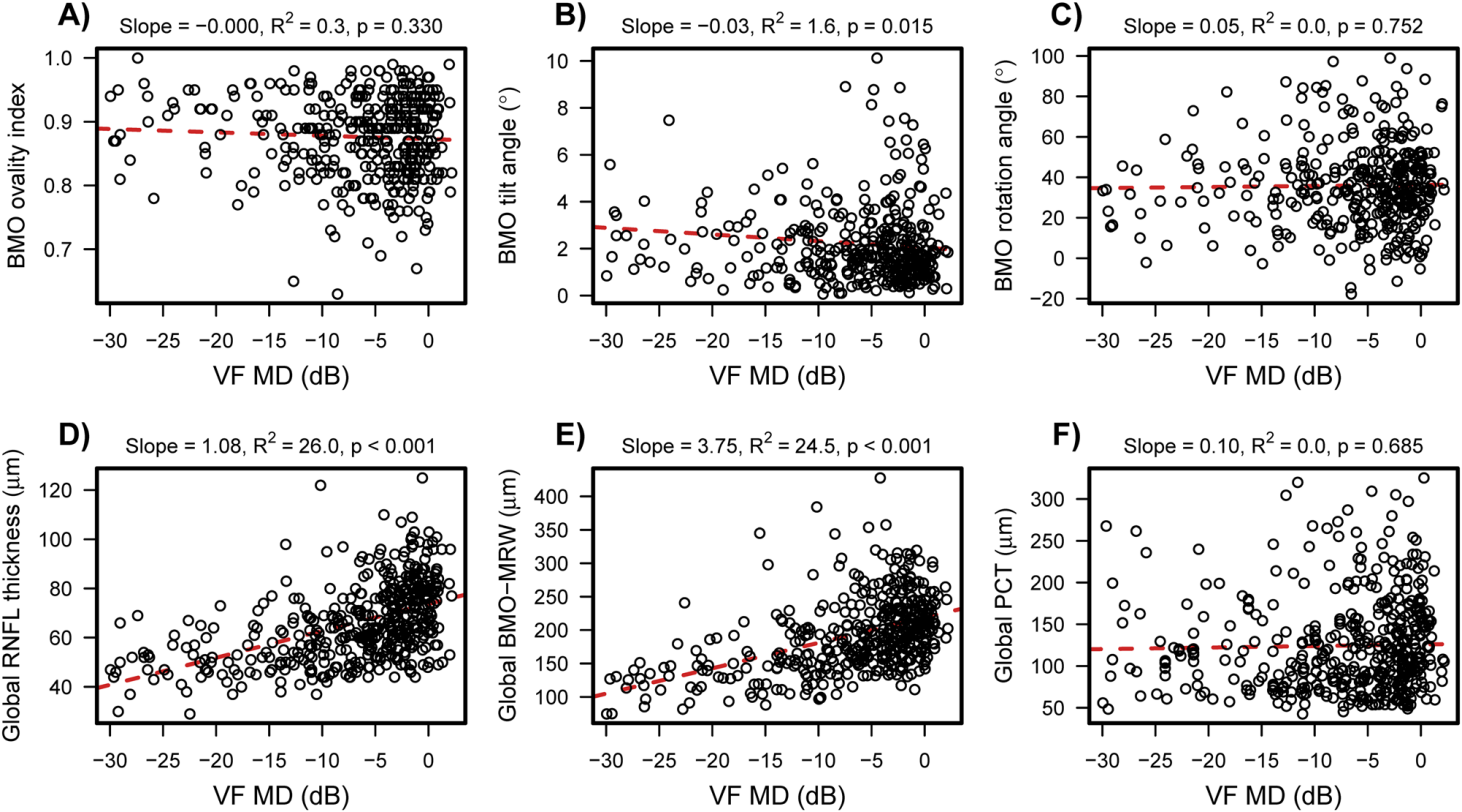
Scatter plots demonstrating association between VF MD and ocular characteristics. Significant associations were found between VF MD and RNFL (**D**) and BMO-MRW (**E**) in multivariate (adjusted for age and axial length) regression analysis. No significant association were found between VF MD and BMO ovality index (**A**), BMO tilt angle (**B**), BMO rotation angle (**C**) and global PCT (**F**), respectively. Abbreviations: BMO, Bruch’s membrane opening; MRW, minimum rim width; PCT, peripapillary choroidal thickness; RNFL, retinal nerve fiber layer; VF MD, visual field mean deviation.

In multivariable regression models adjusted for age and axial length we found global and sectoral RNFL and BMO-MRW to be significantly (semi-partial R^2^) associated with VF MD (Table 4 and Fig. 4) with R^2^ values ranging from 7.3% to 25.9%, and 10.6% to 23.8%, respectively. BMO tilt angle (0.9%, *p* = 0.072) and global (0%, *p* = 0.659) and sectoral PCT (all p > 0.127) were not associated with VF MD in univariate and multivariate regression models.

**Table 4:** The association of ocular characteristics with VF MD.

	N	Univariate regression^a^		Multivariable regression Age and axial length adjusted^a^	
	Patients (Eyes)	Estimate (95% CI)	R^2^ (*p* value)	Estimate (95% CI)	Semi-partial R^2^ (*p* value)
**Age**	277 (452)	0.009 (− 0.007, 0.026)	0.0 (0.267)	0.010 (− 0.007, 0.027)	0.0 (0.248)
**Axial length**	277 (452)	− 0.000 (− 0.009, 0.008)	0.0 (0.921)	− 0.002 (− 0.011, 0.007)	0.0 (0.651)
**BMO morphometrics**	246 (374)				
Ovality index		− 0.001 (− 0.002, 0.000)	0.4 (0.242)	− 0.001 (− 0.001, 0.000)	0.3 (0.307)
Tilt angle (°)		− 0.02 (− 0.05, 0.00)	0.7 (0.100)	− 0.02 (− 0.04, 0.00)	0.9 (0.072)
Rotation angle (°)		0.10 (− 0.21, 0.41)	0.1 (0.517)	0.11 (− 0.20, 0.42)	0.1 (0.489)
**RNFL thickness (µm)**	277 (452)				
Global		1.09 (0.91, 1.26)	25.0 (< 0.001)	1.07 (0.89, 1.25)	23.7 (< 0.001)
Temporal		0.60 (0.40, 0.80)	7.2 (< 0.001)	0.60 (0.40, 0.80)	7.3 (< 0.001)
Superotemporal		1.95 (1.64, 2.26)	26.2 (< 0.001)	1.90 (1.59, 2.21)	24.6 (< 0.001)
Inferotemporal		2.43 (2.05, 2.80)	27.0 (< 0.001)	2.41 (2.03, 2.79)	25.9 (< 0.001)
Nasal		0.60 (0.41, 0.80)	7.9 (< 0.001)	0.59 (0.39, 0.79)	7.4 (< 0.001)
Superonasal		1.09 (0.79, 1.39)	10.6 (< 0.001)	1.05 (0.74, 1.35)	9.6 (< 0.001)
Inferonasal		1.34 (1.05, 1.62)	16.2 (< 0.001)	1.31 (1.02, 1.60)	15.2 (< 0.001)
**BMO-MRW (µm)**	263 (424)				
Global		3.78 (3.13, 4.44)	23.1 (< 0.001)	3.74 (3.08, 4.39)	22.3 (< 0.001)
Temporal		2.29 (1.72, 2.86)	12.3 (< 0.001)	2.24 (1.66, 2.81)	11.7 (< 0.001)
Superotemporal		4.97 (4.15, 5.79)	25.0 (< 0.001)	4.85 (4.03, 5.67)	23.8 (< 0.001)
Inferotemporal		5.43 (4.48, 6.38)	23.0 (< 0.001)	5.36 (4.40, 6.32)	22.1 (< 0.001)
Nasal		3.44 (2.50, 4.39)	10.8 (< 0.001)	3.44 (2.49, 4.39)	10.6 (< 0.001)
Superonasal		4.21 (3.33, 5.09)	17.5 (< 0.001)	4.14 (3.26, 5.02)	16.8 (< 0.001)
Inferonasal		4.89 (3.86, 5.92)	17.6 (< 0.001)	4.86 (3.82, 5.89)	17.3 (< 0.001)
**Choroid thickness (µm)**	249 (399)				
Global		0.27 (− 0.30, 0.85)	0.1 (0.353)	0.13 (− 0.44, 0.69)	0.0 (0.659)
Temporal		0.07 (− 0.65, 0.78)	0.0 (0.856)	− 0.17 (− 0.85, 0.51)	0.0 (0.630)
Superotemporal		0.35 (− 0.38, 1.08)	0.2 (0.346)	0.14 (− 0.57, 0.85)	0.0 (0.705)
Inferotemporal		− 0.14 (− 0.68, 0.39)	0.0 (0.600)	− 0.29 (− 0.81, 0.24)	0.2 (0.286)
Nasal		0.70 (0.02, 1.39)	0.7 (0.046)	0.53 (− 0.15, 1.21)	0.4 (0.127)
Superonasal		0.42 (− 0.33, 1.18)	0.2 (0.275)	0.22 (− 0.52, 0.97)	0.1 (0.557)
Inferonasal		0.16 (− 0.43, 0.76)	0.1 (0.596)	0.01 (− 0.58, 0.60)	0.0 (0.963)

BMO-MRW, Bruch’s membrane opening minimum rim width; CI, confidence interval; ONH, optic nerve head; RNFL, retinal nerve fiber layer; VF MD, visual field mean deviation.

^a^Linear mixed models slope estimates (with 95% confidence intervals) from univariate and multivariable models adjusted for age and axial length.

We found relatively weak associations among the 3 BMO morphometrics. Specifically, BMO ovality index was negatively correlated with the BMO tilt angle (r = −0.23, *p* < 0.001), and was positively correlated with BMO rotation angle (r = 0.16, *p* = 0.007). BMO tilt angle was negatively correlated with BMO rotation angle (r = −0.12, *p* = 0.028).

To validate our results, we also evaluated the association of the BMO parameters with myopia using ovality index, tilt angle and rotation angle calculated by the independent validation method. The independent method showed similar associations between ovality index, tilt and rotation angle and myopic group and axial length as obtained using SALSA described above (data not shown).

## Discussion

This study was unique in that it compared automated OCT-BMO based optic disc characteristics and deep learning assessed PCT of glaucoma eyes with no, mild and high axial myopia with similar severity of glaucoma (based on VF MD) and did not base these measurements on the subjective assessment using a photograph based clinical disc margin. We found that larger BMO tilt angle, lower BMO ovality index and thinner global PCT were associated with increased axial myopia while BMO rotation angle, global and sectoral RNFL thickness and BMO-MRW (except in the temporal, superonasal and inferonasal sectors) were not. This information can be used to inform the clinical management of glaucoma in myopic eyes and can contribute to the understanding of the pathophysiology of the myopic glaucoma eye.

There is evidence that the tilted configuration of the optic disc in myopic eyes is associated with a skewed outlet of the optic nerve from the globe during eyeball elongation^33^. Shearing deformations associated with remodelling of the connective tissue in myopic eyes may also be a factor contributing to the increased tilt^34^. Myopic eyes might therefore be more susceptible to IOP changes due to this anatomic- and tissue biomechanical variation, contributing to higher prevalence of glaucoma among myopic eyes. For this reason, indices that capture this phenomena, such as 1) optic disc ovality index, 2) tilt and 3) rotation have been suggested as possible explanatory factors for the higher prevalence of glaucoma in myopic eyes compared to non-myopic eyes.

Results from previous studies are inconsistent, with some but not all studies reporting an association between axial length and optic disc ovality index^7–9,12,15,16,35^. In most prior studies ovality measurements were based on the clinical disc margin observed in fundus photographs^12–16^. In the current study we utilized custom automated analysis of OCT images to provide objective characterization of glaucoma eyes based on the underlying anatomy across a range of axial lengths. Furthermore, the definition and extent of myopia differed across studies with some reports using spherical equivalent, rather than using the axial length^9,35^. In one of the few studies that included high myopes and used OCT to quantify ovality, Nakanishi et al.^7^ reported an association between axial length and the clinical disc margin based ovality index but not between axial length and the BMO margin based ovality index. Additionally, histological studies have reported the BMO in highly myopic eyes to be circular and not more oval than in non-highly myopic eyes^36^. These results are consistent with our results of no association with BMO based ovality index and axial length.

We also found that objective BMO-based tilt angle was associated with axial length, but not associated with VF MD indicating that larger BMO tilt angles are characteristic of eyes with long axial length and are unrelated to glaucoma. BMO tilt is therefore less likely to be useful to distinguish between healthy and glaucomatous eyes with the same axial length. It is challenging to compare our results to previous findings as the methods for measuring tilt varied across studies. Several studies defined a low ovality index as an indicator for disc tilt, rather than measuring the actual degree angle of the disc tilt^12–14^. Other studies measured optic disc tilt calculated by projecting the clinical disc margin on the OCT ONH height profile and measuring the angle corresponding to where the disc margin and the height profile meet^8,16,18,37^. Park et al. reported that the clinical disc margin from photographs based tilt angle was greater in glaucoma eyes than in healthy eyes with similar axial length, concluding that glaucomatous eyes have more prominent optic disc morphological changes^18^. These definitions of ONH tilt angle are problematic because three-dimensional (i.e. z-axis) information is not utilized.

Utilizing an objective three-dimensional approach, we found rotation angles were similar across the three myopia groups and were not associated with axial length or VF MD. Again, it is challenging to compare results across studies due to methodological differences as the degree of optic disc rotation (often referred to as “torsion”) has been traditionally based on evaluation of two-dimensional clinical fundus photographs by determining the angle between the long optic disc axis and the vertical meridian^7–9,14,16–18^. In this study, we differentiate between “tilt angle” which refers to the optic disc tilt and corresponding BMO rotated around a vertical axis, and “rotation angle” which refers to the rotation around a sagittal axis^38,39^. Optic disc diameters, in particular the long optic disc axis of the optic disc can easily be overestimated in a two-dimensional assessment, because of the slightly oblique view of the optic disc^22^. Studies using the clinical disc margin suggest that the rotation angle is more affected by glaucoma than myopia^8,18^. In contrast to these 2 studies which used two-dimensional definitions of rotation, we found no significant association between three-dimensional BMO rotation angle and VF MD in univariate and multivariate regression analysis. Other authors that applied a three-dimensional approach to assess optic disc rotation angle found the rotation angle to be associated with shorter axial length.

Consistent with our strategy of using the anatomically based BMO ellipse to measure ovality index, tilt and rotation, other studies have also confirmed the importance of utilizing OCT measurements to characterize the optic disc^7,19,22,40^. For instance Dai et al.^22^ demonstrated the discrepancy between the inaccurately short disc diameter measurements (particularly the horizontal disc diameter) by two-dimensional clinical disc margin assessment compared to three-dimensional OCT measurements in myopic and non-myopic eyes. The difference between the two-dimensional and three-dimensional disc diameter measurements increased with increasing axial length, suggesting that disc diameters based on two-dimensional clinical disc measurements may not be accurate for highly myopic eyes^22^.

With excellent performance and reproducibility, OCT assessed RNFL thickness is widely used to detect glaucoma^41,42^. However, it is known that myopia can lead to a significant number of healthy eyes being classified as glaucoma (false-positives) based on comparison to the pattern of RNFL measurements in normative/reference databases of healthy eyes^43–45^. BMO-MRW is proposed as a more stable diagnostic marker to detect glaucoma^23^. Chauhan et al. reported a higher sensitivity at 95% specificity of BMO-MRW compared to RNFL thickness^23^. We found similar global RNFL thickness and BMO-MRW across the three myopia groups, and no significant association with axial length. However, the temporal RNFL thickness increased with increasing axial length and the supra-nasal BMO-MRW sector decreased with increasing axial length. Our results confirm previous reports that the pattern of RNFL thickness and BMO-MRW is different in myopic compared to non-myopic eyes, with myopic eyes often having a temporally located peak RNFL thickness^46,47^. resulting in a higher rate of false positives based on comparisons to normative/reference databases^43,48^. For glaucoma management decisions, RNFL and BMO-MRW values in the temporal and nasal sectors should be interpreted with caution as these sectors show a significant but weak association with axial length.

The role of choroidal vasculature in pathologies such as glaucoma and myopia has been previously investigated^49–52^. According to most clinical studies^49–51,53^ the peripapillary choroidal thickness (PCT) decreases significantly with axial elongation, while an association between axial length and the thickness of the choriocapillaris is still in discussion^54^. As the choroidal vasculature provides the blood supply of the ONH^55^, it is hypothesized that regions with thin choroid are associated with decreased choriocapillaris blood flow and may contribute to the optic disc region being more susceptible to elevated IOP^49,56,57^. We showed a decrease of the PCT with increasing axial length, which is likely due in part to the stretching of choroidal and retinal tissue with axial elongation. Consistent with other studies, we found the inferior sector has the thinnest PCT^49,52,56^. We found global and sectoral PCT to be associated with axial length but not with VF MD, which suggests that thin choroid is rather a characteristic of an axial highly myopic eye than a glaucomatous eye.

Deep learning strategies recently have revolutionized medical and ophthalmic research and enable diseases prediction and image segmentation^58^. We previously measured choroidal thickness using SALSA^59^. The SALSA strategy worked reasonably well, but had a relatively high rate of eyes excluded due to poor segmentation. In the current study we implemented automatized deep learning strategies (SALSA-deep) choroidal layer segmentation in OCT RNFL circle scans. To the best of our knowledge this is the first study utilizing BCDU-Net deep learning-based OCT choroid segmentation in highly myopic glaucoma eyes to assess PCT. SALSA deep did not require a large number of manually segmented scans for its ground truth and resulted in a smaller proportion of eyes excluded due to poor segmentation. A limitation of this approach using RNFL circle scans is that it might not represent choroidal thickness throughout the optic nerve head.

A strength of this study is that we investigated the relationship between axial myopia and optic disc morphology in two ways, by categorizing axial myopia status into three groups and by including axial length as a continuous variable. Both analyses are important. The categorized analysis of axial myopia has the advantage of providing clinicians with clear topographic optic nerve head information regarding the challenging subset of highly myopic eyes, a group whose optic nerve head features are difficult to interpret. Using axial length to define myopia groups instead of using the spherical equivalent, has advantages as it represents better the size of the eye and its axial elongation, then spherical equivalent which changes after cataract and refractive surgery^37^, which is also confirmed by the results in Table 1. However, it should be noted that there are emmetropic eyes, as defined by spherical equivalent, in the mild myopia group. It is therefore important to distinguish between myopia that is defined by refractive error and axial myopia that is defined by axial length and to further keep in mind that spherical equivalent and axial myopia do not correlate strongly in all eyes.

Another study strength is that we used the BMO, an objective anatomical landmark, as the reference plane for analysis of the optic disc morphology instead of the clinical disc margin. Furthermore, we validated the sensitivity of the automated BMO parameters measurements with an independent method and found similar results. In addition, the baseline characteristics of the three glaucoma myopia groups including IOP and the severity of disease (VF MD) were similar across the three groups so that the differences we found among the three groups were more likely due to the different axial lengths and not due to the severity of glaucoma.

Our study includes several potential limitations. First, the mean axial length in the high myopic group was 26.7 mm. We therefore cannot make generalizations about eyes with longer axial length. Second, due to relatively higher prevalence of high myopia in young patients, the high myopia group was significantly younger. For this reason, we incorporated multivariate analysis that adjusted for age. Third, the anatomic features of high myopic optic discs, make accurate BMO segmentation and detection difficult. We therefore meticulously reviewed the BMO segmentation of the radial scans for the BMO-MRW measurement and adjusted the BMO placement accordingly. In addition, to minimize the effect of inaccurate SALSA BMO segmentation, we fit an ellipse to the BMO plane for the custom calculation of ovality index, tilt and rotation. Fourth, myopia was defined by axial length but the axial length measurement was not repeated and evaluated for reproducibility. However, previous studies have reported that IOLMaster measurements show good repeatability and accuracy for axial length assessment^60^.

In summary, using objective and anatomically based OCT measurements, we found that glaucoma eyes with highly myopic optic discs were characterized by larger BMO tilt and smaller PCT, and these parameters were not associated with severity of glaucoma (visual field mean deviation). Furthermore, caution should be exercised when using sectoral BMO-MRW and RNFL thickness for glaucoma management decisions in myopic eyes as the pattern varies with axial length.

## Supplementary Information

Supplementary Information 1.

Supplementary Information 2.

Supplementary Information 3.

## Data Availability

The data that support the findings of this study are available from the corresponding author upon reasonable request.
