# Supplementary figures and images for: The influence of axial myopia on optic disc characteristics of glaucoma eyes
Source: Sci Rep. 2021 Apr 23;11:8854. doi: 10.1038/s41598-021-88406-1 (PMC8065167; doi:10.1038/s41598-021-88406-1)

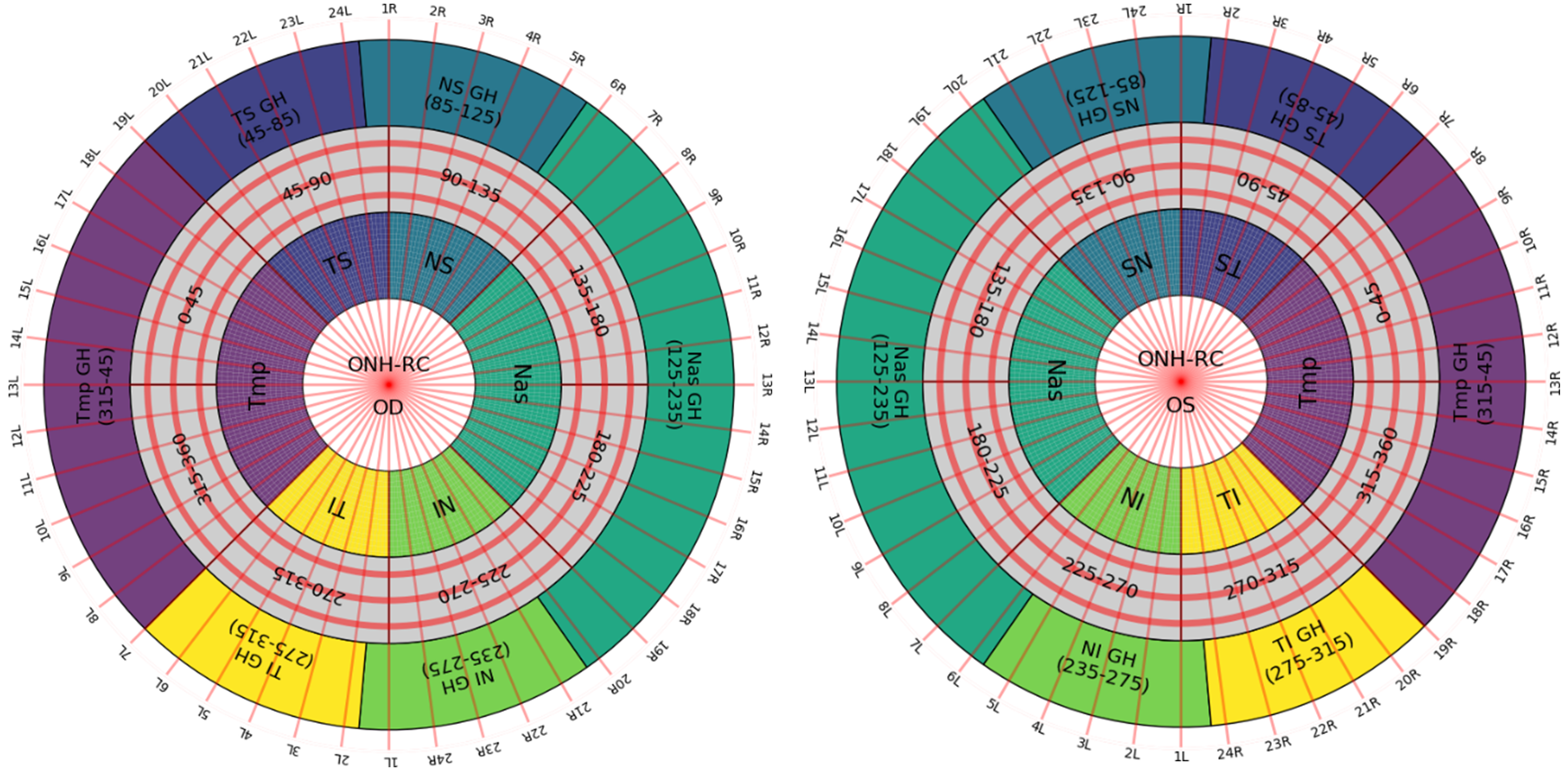

Supplement: Supplementary file 1 — Supplementary Information 1. [file 41598_2021_88406_MOESM1_ESM.tif]

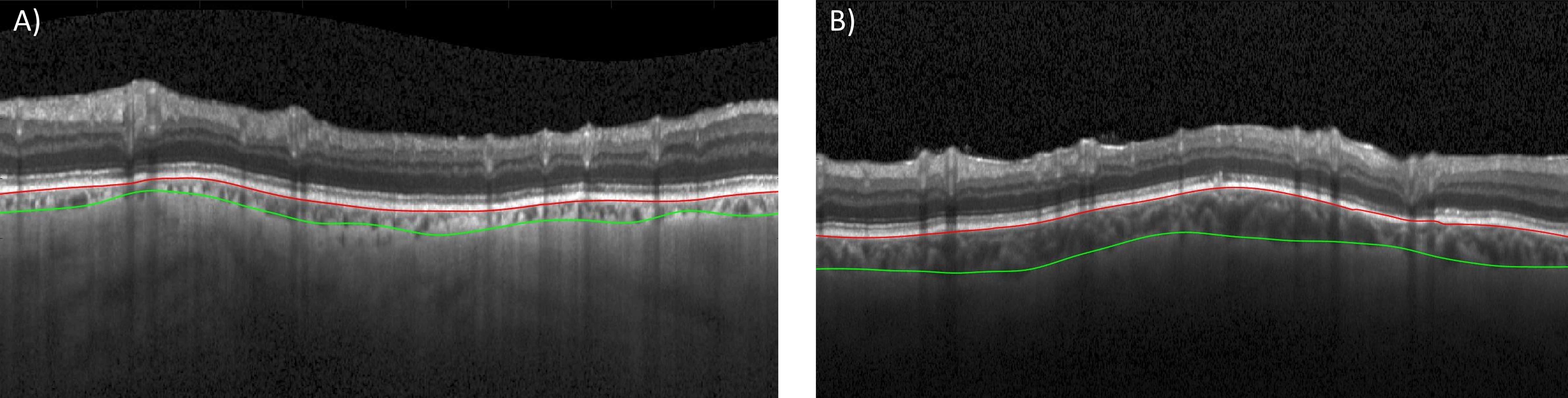

Supplement: Supplementary file 2 — Supplementary Information 2. [file 41598_2021_88406_MOESM2_ESM.tif]
