# Supplementary material for: The influence of axial myopia on optic disc characteristics of glaucoma eyes
Source: Sci Rep. 2021 Apr 23;11:8854. doi: 10.1038/s41598-021-88406-1 (PMC8065167; doi:10.1038/s41598-021-88406-1)
Supplement: Supplementary file 3 — Supplementary Information 3. [file 41598_2021_88406_MOESM3_ESM.docx]

The Influence of Axial Myopia on Optic Disc Characteristics of Glaucoma Eyes

Jasmin Rezapour^1,2^, Christopher Bowd^1^, Jade Dohleman^1^, Akram Belghith^1^, James A. Proudfoot^1^, Mark Christopher^1^, Leslie Hyman^3^, Jost B. Jonas^4^, Massimo A. Fazio^5,6^, Robert N. Weinreb^1^, Linda M. Zangwill^1,*^

1 Hamilton Glaucoma Center, Shiley Eye Institute, Viterbi Family Department of Ophthalmology, UC San Diego, La Jolla, CA, United States

2 Department of Ophthalmology, University Medical Center of the Johannes Gutenberg University Mainz, Germany

3 Wills Eye Hospital, Thomas Jefferson University, Philadelphia, PA, United States

4 Department of Ophthalmology, Medical Faculty Mannheim, Heidelberg University, Mannheim, Germany

5 Department of Ophthalmology and Vision Science, School of Medicine, The University of Alabama at Birmingham, Birmingham, AL, United States

6 Department of Biomedical Engineering, School of Engineering, The University of Alabama at Birmingham, Birmingham, AL, United States

*Corresponding author:

Linda M. Zangwill

9500 Gilman Drive

La Jolla, CA 92093-0946

Shiley Eye Institute/Hamilton Glaucoma Center

Viterbi Family Department of Ophthalmology

University of California, San Diego

T: (858) 534-7686

Email: [lzangwill@health.ucsd.edu](mailto:lzangwill@health.ucsd.edu)

Running Head: Optic Disc Parameters in Glaucoma Eyes with Axial Myopia

**Supplemental Material**

**BMO ovality, tilt and rotation calculation**

Optic disc ovality, tilt and rotation parameters were calculated on the segmented radial B-scans of the optic nerve head radical circle (ONHRC) scans from raw image export (.vol) files. Each b-scan was processed using the Python-based San Diego Automated Layer Segmentation Algorithm (SALSA)-Image Processing Pipeline. Details of SALSA and its validation have been described previously.^1-3^

The 4 points (2 Bruch’s membrane border [BMB] and 2 Bruch’s membrane opening [BMO] points) for each of the 24 ONH radial B-scans were used to model the optic nerve head and calculate the BMO ovality index, tilt and rotation of the fitted ellipse (Figure 1A Main Manuscript). All computations were performed using 3D rectangular coordinates (Cartesian system). Each b-scan has a unique transformation from the cylindrical space of the scan volume to the rectangular space. This coordinate transformation was determined by the b-scan index and eye (right or left). The sectoral mapping of these transformations and sector definitions is shown in Supplemental Figure 1. Each point from both the BMB segmentation and the BMO segmentation is transformed into the rectangular coordinate system (Figure 1B Main Manuscript). Angles are reported after transforming the vectors used in their computations into physical space by scaling according to the scale x and scale z constants in the image volume metadata. All measurements were provided with respect to individual anatomies by factoring in the Fovea-BMO center (FoBMOc) angle captured by the Spectralis OCT.

**Details of BDCU-Net implementation for segmentation of choroid from RNFL circle scans**

U-nets, a type of fully convolutional neural network have been utilized increasingly for automated image classification, and semantic segmentation.^4,5^ An advantage of U-net architecture is that it works well with limited training samples by utilizing the global location and context information at the same time. The network consists of two steps: the convolutional encoding and decoding steps. The convolution operations are performed followed by ReLU activation in both parts of the network. In addition, 2×2 max-pooling operations are performed for down sampling. In the decoding phase, the convolution transpose is performed to up-sample the feature maps. The end-to-end pipeline processes the entire image in the forward pass and directly produces segmentation maps.

Recently, others have shown better performance than state-of-the-art alternatives for segmentation tasks using a new approach BCDU-Net, an extension of U-Net, bi-directional ConvLSTM^6^ and the dense convolutions approach.^7^ Specifically, BCDU-net combines the features obtained from the corresponding encoding path and the previous decoding up-convolutional layer instead of the simple concatenation approach used in the standard U-Net. Moreover, the densely connected convolutions were used in the last convolutional layer of the encoding path to strengthen feature propagation and enable feature reuse.

To segment the choroid on the RNFL circle scans by identifying the BM and the posterior boundary of the choroid (Supplemental Figure 2) we applied the BCDU-Net approach, utilizing Keras with TenserFlow as the backend. The network has been trained from scratch. We used the ADAM optimization technique with a learning rate of 2 × 10−4 and binary cross-entropy loss. We stopped the training of the network when the validation loss remainsed the same in 5 consecutive epochs.

**Supplemental Figure Captions**

**Supplemental Figure 1:** Sectoral map for left (OS) and right (OD) eyes of b-scans by number (1-24) and image side (R=right, L=left).

Circular red lines represent the circular scans and radial red lines the radial scans. Angle distribution from 0° to 360° mapped on the inner circle. Two sector definitions are shown. The Garway-Heath (GH) sectors are defined outside the RNFL circle and the standard Spectralis ONH sectors defined inside the RNFL circle.

Abbreviations: IN, inferonasal; Nas, nasal; NS, superonasal; ONH, optic nerve head; RC, radial circle; Tmp, temporal; TS, superotemporal

**Supplemental Figure 2:** Deep learning assessed peripapillary choroidal thickness (PCT) in an optic nerve head OCT radial circle scan of (A) a highly myopic eye with axial length of 26.1mm and PCT of 52.0µm and (B) a non-myopic eye with axial length of 23.2mm and PCT of 133.8µm of similar age (55 and 50 years, respectively) and visual field MD (-5.5 dB and -5.0 dB, respectively).

PCT is measured between the red and green line. The red line indicates the Bruch’s membrane and the green line the posterior boundary of the choroid.

**References**

1. Belghith A, Bowd C, Medeiros FA, et al. Does the Location of Bruch's Membrane Opening Change Over Time? Longitudinal Analysis Using San Diego Automated Layer Segmentation Algorithm (SALSA). Investigative ophthalmology & visual science 2016;57:675-82.

2. Mundae RS, Zangwill LM, Kabbara SW, et al. A Longitudinal Analysis of Peripapillary Choroidal Thinning in Healthy and Glaucoma Subjects. American journal of ophthalmology 2018;186:89-95.

3. Belghith A, Bowd C, Weinreb RN, Zangwill LM. A hierarchical framework for estimating neuroretinal rim area using 3D spectral domain optical coherence tomography (SD-OCT) optic nerve head (ONH) images of healthy and glaucoma eyes. Conference proceedings : Annual International Conference of the IEEE Engineering in Medicine and Biology Society IEEE Engineering in Medicine and Biology Society Conference 2014;2014:3869-72.

4. Long J, Shelhamer E, Darrell T. Fully convolutional networks for semantic segmentation. Proceedings of the IEEE conference on computer vision and pattern recognition, 2015:3431-3440.

5. Ronneberger O, Fischer P, Brox T. U-net: Convolutional networks for biomedical image segmentation. International Conference on Medical image computing and computer-assisted intervention: Springer, 2015:234-241.

6. Song H, Wang W, Zhao S, Shen J, Lam K-M. Pyramid dilated deeper convlstm for video salient object detection. Proceedings of the European Conference on Computer Vision (ECCV), 2018:715-731.

7. Huang G, Liu Z, Van Der Maaten L, Weinberger K. Densely connected convolutional networks In: Proceedings of the IEEE Conference on Computer Vision and Pattern Recognition, 2017.
